# SH2db, an information system for the SH2 domain

**DOI:** 10.1093/nar/gkad420

**Published:** 2023-05-19

**Authors:** Dávid Bajusz, Gáspár Pándy-Szekeres, Ágnes Takács, Elvin D de Araujo, György M Keserű

**Affiliations:** Medicinal Chemistry Research Group and National Laboratory for Drug Researchand Development, Research Centre for Natural Sciences, Magyar tudósok krt. 2, 1117 Budapest, Hungary; Medicinal Chemistry Research Group and National Laboratory for Drug Researchand Development, Research Centre for Natural Sciences, Magyar tudósok krt. 2, 1117 Budapest, Hungary; Department of Drug Design and Pharmacology, University of Copenhagen, Universitetsparken 2, 2100 Copenhagen, Denmark; Medicinal Chemistry Research Group and National Laboratory for Drug Researchand Development, Research Centre for Natural Sciences, Magyar tudósok krt. 2, 1117 Budapest, Hungary; Centre for Medicinal Chemistry, University of Toronto at Mississauga, Mississauga, ON L5L 1C6, Canada; Medicinal Chemistry Research Group and National Laboratory for Drug Researchand Development, Research Centre for Natural Sciences, Magyar tudósok krt. 2, 1117 Budapest, Hungary; Department of Organic Chemistry and Technology, Faculty of Chemical Technology and Biotechnology, Budapest University of Technology and Economics, Műegyetem rkp. 3, 1111 Budapest, Hungary

## Abstract

SH2 domains are key mediators of phosphotyrosine-based signalling, and therapeutic targets for diverse, mostly oncological, disease indications. They have a highly conserved structure with a central beta sheet that divides the binding surface of the protein into two main pockets, responsible for phosphotyrosine binding (pY pocket) and substrate specificity (pY + 3 pocket). In recent years, structural databases have proven to be invaluable resources for the drug discovery community, as they contain highly relevant and up-to-date information on important protein classes. Here, we present SH2db, a comprehensive structural database and webserver for SH2 domain structures. To organize these protein structures efficiently, we introduce (i) a generic residue numbering scheme to enhance the comparability of different SH2 domains, (ii) a structure-based multiple sequence alignment of all 120 human wild-type SH2 domain sequences and their PDB and AlphaFold structures. The aligned sequences and structures can be searched, browsed and downloaded from the online interface of SH2db (http://sh2db.ttk.hu), with functions to conveniently prepare multiple structures into a Pymol session, and to export simple charts on the contents of the database. Our hope is that SH2db can assist researchers in their day-to-day work by becoming a one-stop shop for SH2 domain related research.

## Graphical Abstract

**Graphical Abstract:**
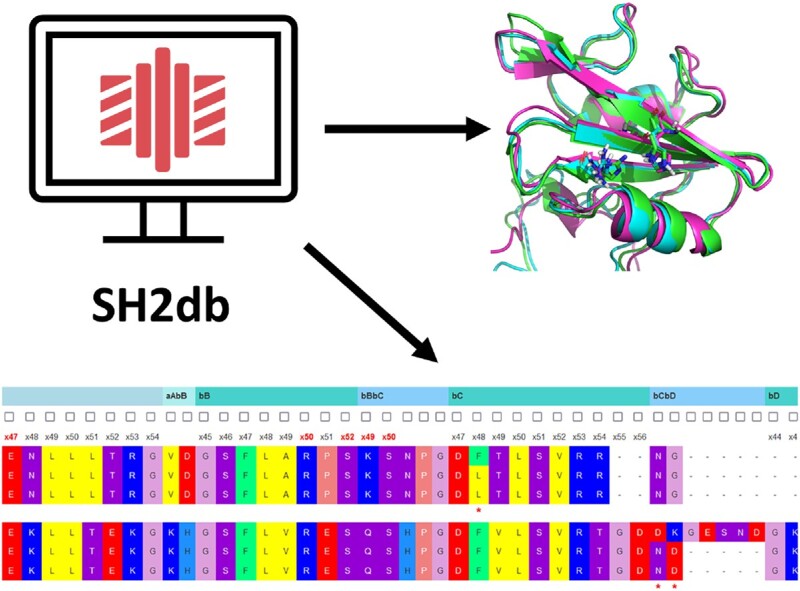

## INTRODUCTION

The Src homology 2 (SH2) domain was one of the first protein-protein interaction (PPI) modules to be discovered (1). By recognizing specific phosphotyrosine (pTyr)-containing peptide motifs, this small (approx. 100 amino acids) protein module acts as the reader unit of pTyr-based signal transduction, an intracellular signaling system that emerged about 600 million years ago, just prior to multicellular organisms (2). In addition to SH2 domains, this signaling system employs protein tyrosine kinases (PTK) as writer units, and pTyr phosphatases (PTP) as eraser units (3). In accordance with the ubiquity of pTyr signaling in eukaryotic cells, it was realized early on that the disruption of this signaling system by protein mutations or pathogens contributes to a range of disease conditions (4,5), with the Src and Grb2 SH2 domains as early examples of therapeutic proteins being targeted by small-molecule and peptide inhibitors (6). From the very beginning, SH2 domains have been regarded as challenging drug targets, due to the shallow binding surface that is characteristic of protein-protein interactions in general (7). Nonetheless, the therapeutic interest in targeting SH2 domains has steadily grown with the abundant discoveries of disease-causing mutations in a wide range of SH2 domains (8–11). In the meantime, a large body of SH2-related computational and experimental know-how has been accumulated (12).

A total of 120 SH2 domains are present in 110 human proteins – ten of which contain dual SH2 domains. Over the years, several approaches have been employed to group these proteins into informative sub-classes (13,14), with the most prominent strategies based on collecting the SH2 domains into 11 functional categories by Liu and colleagues (15). Interestingly, this functional grouping does not correlate directly with the phylogenetic distances of the SH2 domains themselves, although we have recently found that sequence similarities within these functional categories are higher if we account for the general character (polar, aromatic, etc.) of the amino acid sidechains (16). For the past two decades, the main structural features of the SH2 domain have been well understood: its centerpiece is an antiparallel β-sheet (with three strands labelled βB-βD) sandwiched between two α-helices (αA and αB), also referred to as the αβββα motif (10). The central β-sheet divides the binding surface of the SH2 domain into two subpockets, called the phosphate-binding (pY) and specificity (pY + 3) pockets. Upon phosphopeptide binding, the β-sheet is perpendicularly bridged by the interacting partner, exposing its phosphotyrosine group against an (almost) invariant arginine residue on the βB strand, while neighbouring sidechains in the C-terminal direction (labelled + 1, +2, etc. from the phosphotyrosine residue) are recognized by the specificity pocket (17). The Sheinerman residues, a group of eight residues in the pY pocket (including the critical arginine) are primarily responsible for anchoring the phosphotyrosine group (18), and their mutations are usually detrimental to SH2 domain function (10). A short, conserved sequence of residues within the βB strand defines the so-called ‘SH2 signature motif’ (19), also known as the FLXRXS or FLVR motif, which includes the critical arginine residue. Interestingly, there are a small number of proteins (RIN2, TYK2 and SH2D5 in humans) where this arginine is replaced by an aromatic residue: these SH2 domains recognize acidic residues other than pTyr (Glu or Asp) in non-typical binding modes (20). With a fairly robust understanding of the typical functions and structural features of SH2 domains, recent studies were directed to more specific questions, such as posttranslational modifications other than phosphorylation (21), the role of water molecules in phosphopeptide binding (22), development of SH2 superbinders (23), or simultaneous phosphotyrosine binding in a protein with dual SH2 domains (24).

Structural databases boost the productivity of computational medicinal chemists and modelers by offering highly specialized and relevant information on protein families of high interest and therapeutic relevance. A prominent example is GPCRdb, a database of G-protein coupled receptor (GPCR) structures, sequences and ligands, published in its modern form in 2014 (25), maintained and regularly updated by the Gloriam group (https://gpcrdb.org/). GPCRdb contains a range of useful features, including generic residue numbers for the convenient comparison of residue positions in different proteins (26), integration of mutagenesis data (27), annotation of different functional types of ligands (28), or as its latest addition, the incorporation of AlphaFold (29) models of GPCRs (30). Similarly, the Kinase–Ligand Interaction Fingerprints and Structure database (KLIFS) was introduced in 2014 for the convenient mining of the available structural information on kinase inhibitors and their interaction patterns (31), with its functionality expanded multiple times (32). For SH2 domains, such a convenient and up-to-date online resource is missing as of yet: while an earlier database from the Nash and Pawson labs is still available online (https://sites.google.com/site/sh2domain/home), this mostly focused on providing links to major sequential and structural databases (Entrez, UniProt, PDB, etc.), and was not updated since 2015. We should also point to a few, more generic databases that are useful in the research of SH2 domains, including Phospho.ELM for referencing experimentally validated phosphorylation sites (33,34), and Scansite for searching for potential interacting partners of SH2 domains (35).

Here, we outline the development, architecture and main functionalities of SH2db, a database and webserver for SH2 domain sequences and structures. With SH2db, our aim is to provide a convenient starting point to bioinformaticians, computational and medicinal chemists, and practitioners of related fields for any studies where they utilize SH2 domain structures. In particular, we have revised the sequence alignment of human SH2 domains published by Liu et al. (15), introduced a generic residue numbering scheme for the comparability of residue positions in different SH2 domains, and launched a webserver to facilitate quick access to any arbitrary sets of pre-aligned SH2 domain sequences (*fasta* format) or structures (*pdb* format or *Pymol* session). Experimental and theoretical protein structures have been incorporated into SH2db from the PDB (36) and AlphaFold databases (29,37). The SH2db webserver is available at http://sh2db.ttk.hu/, while its source code is shared at https://github.com/keserulab/SH2db.

## MATERIALS AND METHODS

### Data

Protein sequences were retrieved from UniProt (38), experimental structures were downloaded from the Protein Data Bank (PDB) (36,39) and AlphaFold models were gathered from the EMBL-EBI AlphaFold repository (29,40). The PDB files were parsed, renumbered to match the wild-type sequence and non-SH2 domain parts were removed. Structures containing two SH2 domains or the same domains in several chains were split into separate PDB files. In this first release of SH2db, we included only human sequences with their canonical isoform, but built the framework to allow easy incorporation of ortholog sequences and other isoforms in the future.

### Framework

SH2db uses the python-based Django framework with the PostgreSQL object-relational database system. The hierarchy of the database starts on two parallel top levels, Protein and Structure, which both link to lower levels of objects: Protein–Isoform–Protein domain; Structure–Chain–Structure domain. We store the wild-type protein-related data (species, sequence, protein family, IDs) in the Protein hierarchy and structure-related data in the Structure hierarchy (PDB data, publication, experimental method, resolution, IDs). The two hierarchies are connected on the top level and also on the Protein domain–Structure domain level. This latter connection is the main driver of the online tools as these objects store the SH2 domain units that are listed in several pages. Also, Residue objects are linked to the Protein domain objects, which powers the sequence alignments. Protein segment and Generic number objects are connected to Residue objects. AlphaFold models are linked to wild-type Protein objects.

### Generic residue numbering

**Figure 1.**
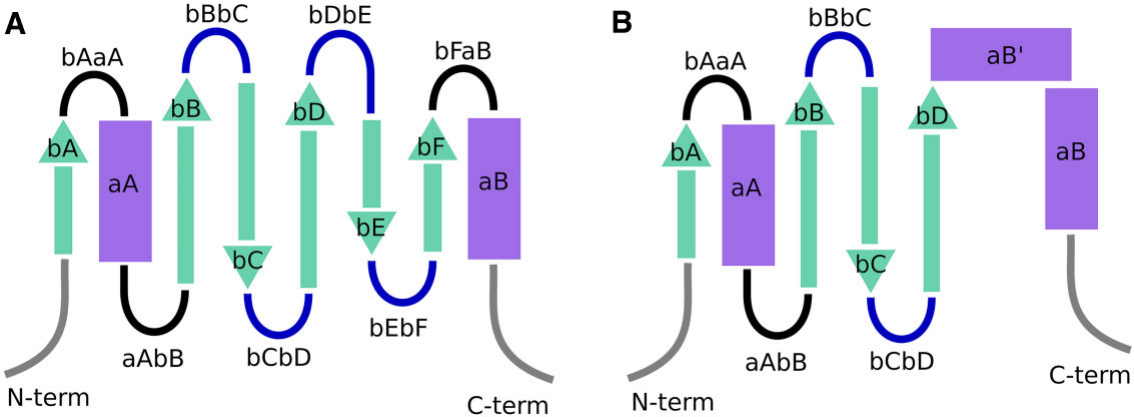
Schematic representation of the structurally conserved segments of SH2 domains. Grey represents the terminals, black disordered loops, blue turns between strands, turquoise beta strands and purple alpha helices. Generic numbered positions are assigned to all beta strands and alpha helices. (**A**) In the most common segment layout of SH2 domains, strand bD is followed by two shorter strands bE and bF, then the domain ends with the aB helix, with all of them connected by loops or turns. (**B**) In the STAT proteins, the bE and bF strands with their flanking loops/turns are missing and instead the aB’ helix is present, which connects to bD and aB without any loops or turns.

**Figure 2.**
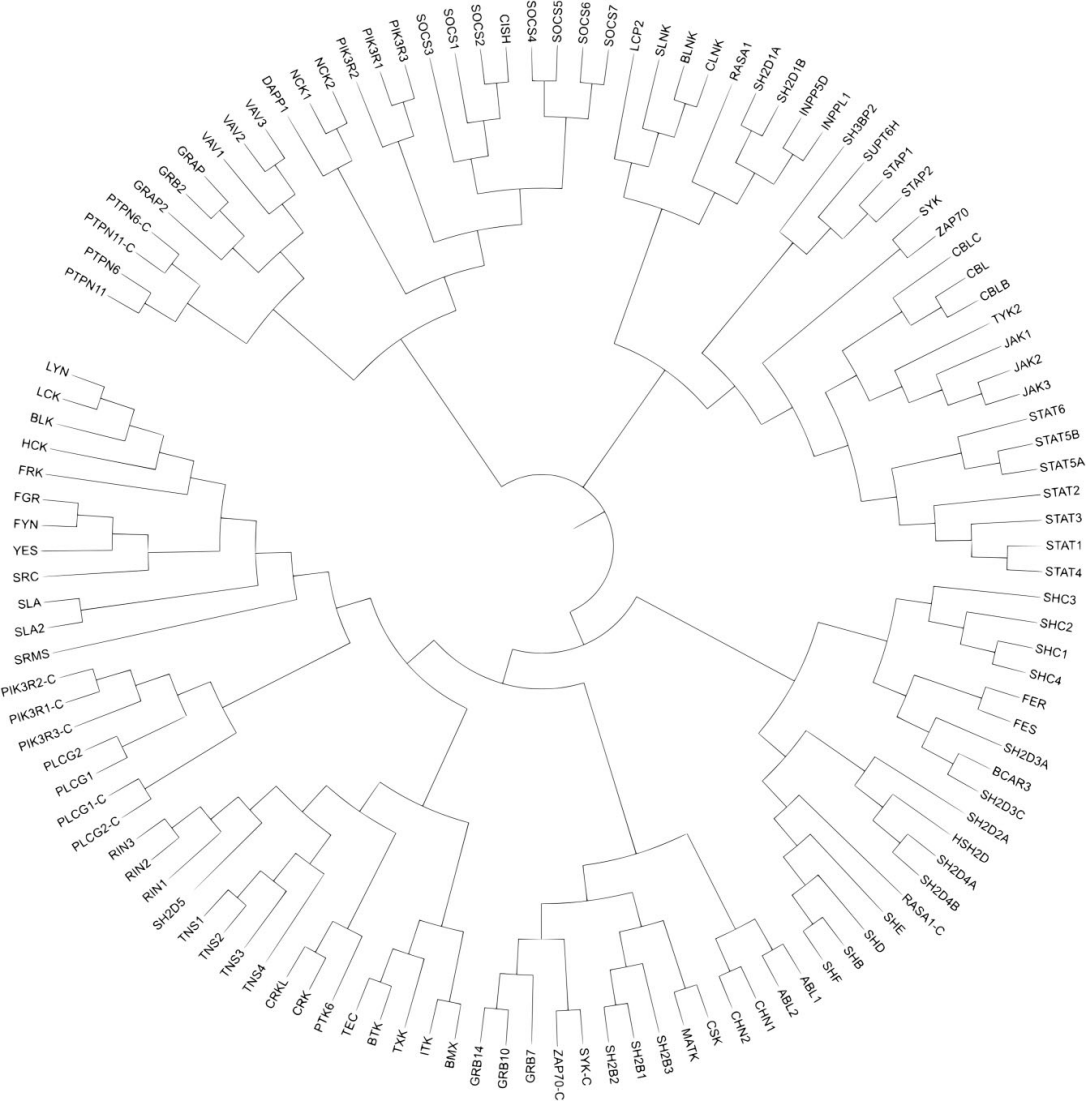
Phylogenetic tree of the SH2 domain containing proteins using the sequence alignment of positions with a generic number. The ‘-C’ tag denotes the C-terminal SH2 domain (in proteins with two SH2 domains). The tree was made with Biopython's Phylo module and iTOL (60).

Similarly as done for GPCRs with the Ballesteros-Weinstein (41) or the GPCRdb generic numbering scheme (26), we aimed to develop generic residue numbers for the SH2 domain to easily perform structure and sequence based comparisons between members of the family. An initial structural superposition was performed on all structures in Schrödinger's Maestro (Schrödinger Release 2022-4: Maestro, Schrödinger, LLC, New York, NY, 2022). Starting out from the multiple sequence alignment of Liu *et al.* (15), we made local structure-based alignments and adjusted the sequence alignment accordingly. Mainly focusing on the segments with conserved secondary structural characteristics, we were able to determine the most conserved residue positions throughout the human sequences. In each segment with a conserved secondary structural characteristic, the most conserved position was labeled as ‘×50’, while residues in either direction that belong to this same segment were labeled sequentially. We identified and numbered three α-helices (aA, aB’ and aB) and six β-strands (bA, bB, bC, bD, bE and bF). In addition, we assigned two generic numbers to two Sheinerman residues that are located in the bBbC turn. Loops in between the helices and strands were labelled based on the flanking segment labels (e.g. the loop between bA and aA is bAaA). Due to their disordered and flexible nature, we opted not to give generic numbers to the loops as structure-based comparison is not possible for many of these segments, due to the corresponding residues not occupying the same 3D space. Importantly, helix aB’ is exclusively found in the SH2 domains of the STAT protein family, and has been referred to as the Evolutionary Active Region (13) in SH2 domains (Figure 1). Based on the sequence alignment from the numbered positions, we created a phylogenetic tree to showcase the evolutionary distances between SH2 domain containing proteins (Figure 2).

### Superposition

After multiple iterations of structural alignment, we found that superposing the backbone atoms of residues from the core β-sheet (comprised of β-strands bB, bC and bD) yielded the most reliable overlay for the whole set. All structures and models available on SH2db were superposed based on these residues using the structure of the FER kinase (PDB: 2KK6) as reference, running the ‘align’ function of Pymol (The PyMOL Molecular Graphics System, Version 1.9.0.0 Schrödinger, LLC). These superposed structures are exposed to all of the download functions, including an internal script for generating Pymol sessions on-the-fly for download. (On the website, a brief message informs the user about the licensing options of Pymol.)

## RESULTS

We have engineered a webserver that currently stores 352 PDB and 120 AlphaFold structures of human SH2 domains in a preprocessed and pre-aligned fashion, and provides simple and intuitive interfaces for searching, filtering and downloading arbitrary sets of the underlying data in multiple formats. The webserver and the underlying database were built in the spirit of scalability, implementing a hierarchy of Django data models (and corresponding PostgreSQL database fields) that allow for significant extensions later on, e.g. the addition of SH2 domains from diverse species, providing additional links to external databases, incorporating isoforms, etc.

**Figure 3.**
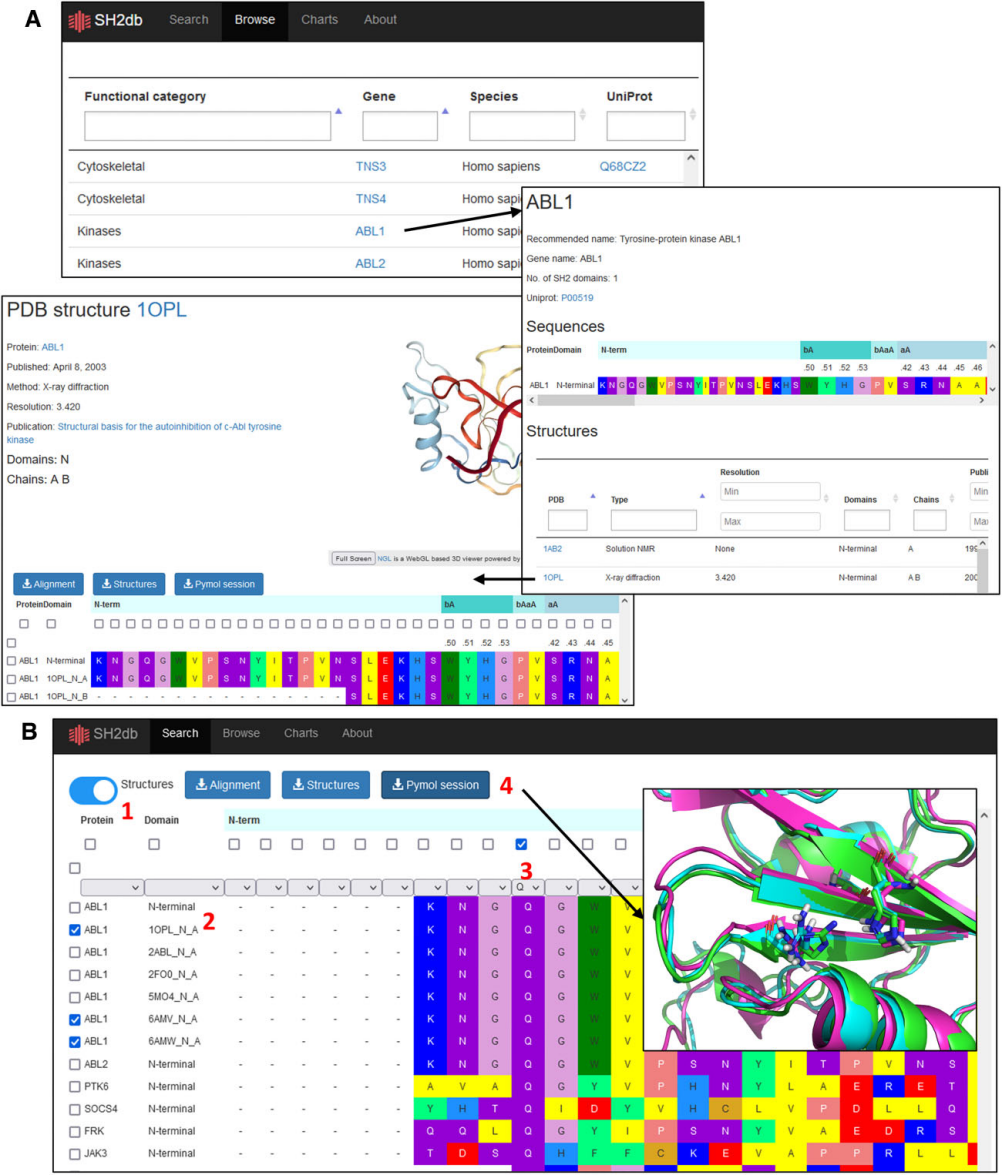
SH2db provides two main ways for accessing the underlying database. (**A**) From the Browse page, the user can hierarchically navigate first to entries of specific proteins, and then to specific structures. The entries contain external database links, a sequence viewer, download options and an interactive NGLviewer panel for quick visualization. (**B**) The Search page provides functionalities to filter the underlying database via an interactive sequence viewer and download arbitrary selections of sequences or structures. The toggle button (1) switches between including structure entries or restricting the table to canonical protein sequences extracted from UniProt. The Domain column (2) lists the PDB IDs or marks the UniProt sequences and AlphaFold models by their Uniprot ID, followed by ‘N’ or ‘C’ for proteins with dual SH2 domains (or ‘N’ by default for single-SH2 proteins). The table can be filtered by any combination of fields, including individual amino acid positions (3). Selections can be downloaded as sequences (fasta), structures (pdb) or fed into a backend script to generate a Pymol session (4), which shows the selected structures superposed, and the selected residues highlighted as sticks, for a quick and easy structure comparison.

In its first published version, the SH2db webserver (available at http://sh2db.ttk.hu/) provides access to SH2 domain sequences, structures and models in two main ways (Figure 3). From the Browse page, the user can access a hierarchy of individual database entries, presented on informative summary pages. Protein entries link to their corresponding UniProt page, feature a sequence viewer showing the canonical sequence, and a table that summarizes, and links to, the corresponding structure and model entries, along with core information on experimental/modeling method, resolution, etc. Structure entries link to their respective PDB entry, publication, feature an interactive sequence viewer with options for downloading, and an interactive NGLviewer panel for quick visualization.

The Search page offers an alternative route: by starting from a large, interactive sequence viewer, the user can select an arbitrary set of sequences, structures and residues, to be exported into a fasta file, a set of pdb files or, using a backend script, a pre-formatted Pymol session. The Pymol session features the selected structures superposed, and the selected residues saved in named selections and highlighted in stick representation. The AlphaFold models are linked to the wild type sequence of the SH2 domain and labeled *<UniProt accession>-AF-<domain type >* e.g. *Q13191-AF-N* for the AlphaFold model of the N-terminal SH2 domain of CBLB. The miscellaneous pages offer quick visual summaries of the current contents of SH2db (Charts) and explanatory texts on the main features of SH2 domains, and SH2db itself (About and Documentation).

In the following subsections, we aim to demonstrate the key features of SH2db in two short case studies of structural comparisons. In both cases, it takes the user only a few minutes of browsing the database and a few clicks to produce a script-generated Pymol session that provides a convenient starting point for comparing SH2 domain structures. By downloading the pre-aligned pdb structures, the user can submit molecular dynamics simulations, binding site analysis and virtual screening or other modeling jobs for multiple SH2 structures that will be easy to compare upon completion.

### Case study 1: effect of the N642H mutation on the peptide binding affinity of the STAT5B SH2 domain

Signal transducers and activators of transcription (STAT) are a family of seven multidomain transcription factors with key roles in intracellular signaling, primarily in the JAK/STAT signaling pathway (42). STAT proteins, especially STAT3 and STAT5B have been identified as potential pharmaceutical targets in a range of oncological conditions, including various types of leukemias and solid cancers (43–45). STATs are multidomain proteins that can enter the nucleus and initiate gene transcription upon parallel (active-state) dimer formation via their SH2 domains, following phosphorylation at a conserved tyrosine residue (46). In this context, the SH2 domain thus acts as a mediator of dimer formation, by recognizing the tyrosine-phosphorylated, C-terminal tail segment of the opposing STAT monomer. In addition to its importance as a direct pharmaceutical target, the SH2 domain is a hotspot for a variety of oncogenic mutations in STAT3 and STAT5B, which are direct drivers of disease conditions, with their exact structural impact only partially understood as of yet (10).

**Figure 4.**
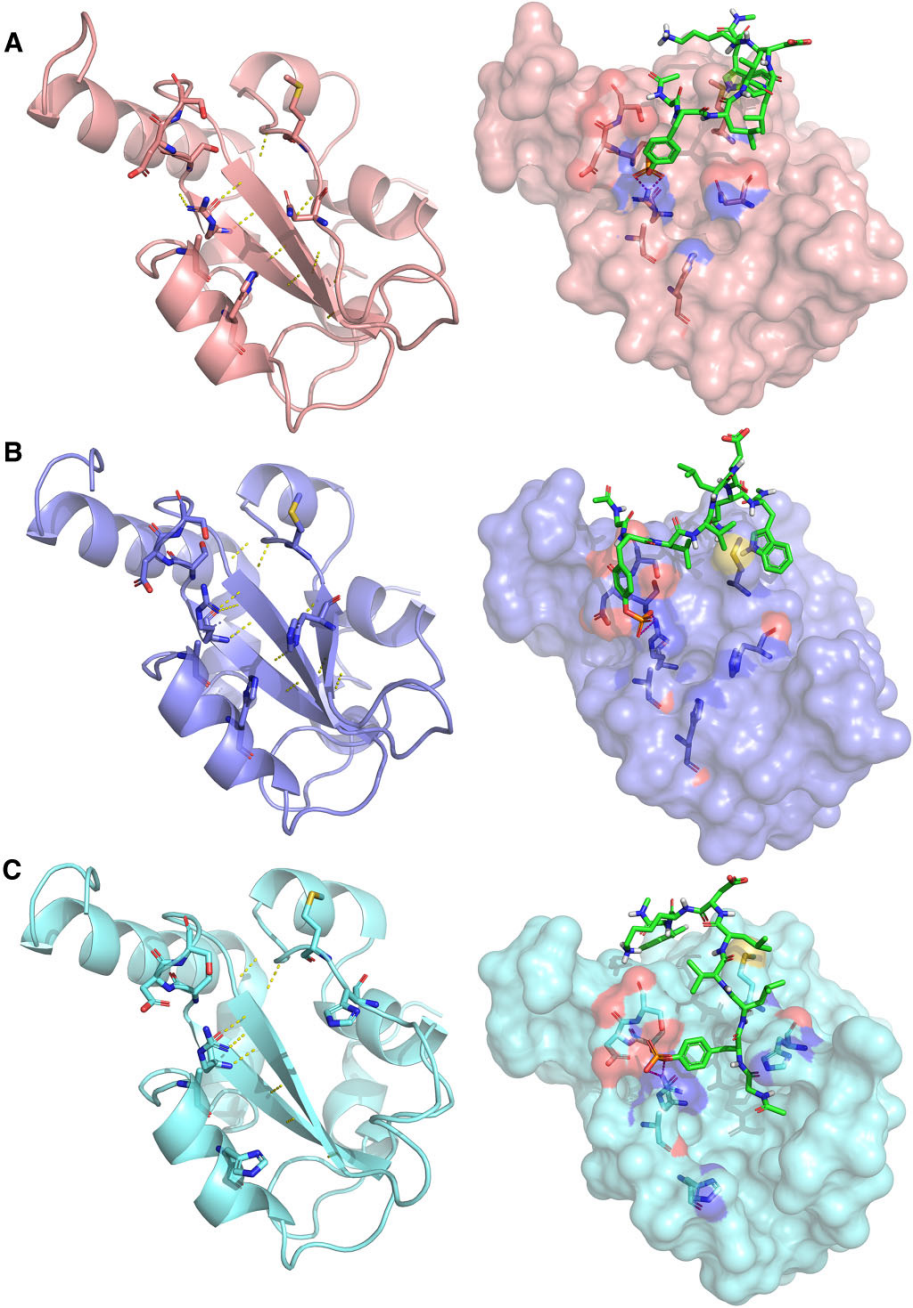
Structures (left) of the wild-type (A, PDB: 6MBW), N642H ‘tight-bD’ (B, PDB: 6MBZ, chain B) and ‘loose-bD’ (C, PDB: 6MBZ, chain A) conformation STAT5B SH2 domains (49), and the docking poses of the GpYLVLDKW peptide (green) against these domains (right). In the ‘loose-bD’ conformation, the dissociated bD strand contributes to the formation of a small subpocket that can accommodate the N-terminal end of the phospho-peptide within the pY pocket.

Recently, the X-ray structures of the STAT5B SH2 domain, as well as its oncogenic N642H mutant were solved (47). Interestingly, the authors have simultaneously identified two distinct conformational states for the mutant SH2 domain: in one of them, the bD strand forms additional hydrogen bonds with the bC strand, as compared to the wild-type structure (Figure 4B, we will refer to this as ‘tight-bD’ conformation from here on). The other conformation presents a dissociated bD strand (‘loose-bD’ from here on), and thereby a greater structural difference from the wild-type SH2 domain (Figure 4C). In addition to solving the crystal structures, the authors have determined, via a fluorescence polarization assay (48), that the N642H mutation increases the binding affinity of the fluorescently labeled phosphopeptide GpYLVLDKW (derived from the EPO receptor) by about 7-fold. However, the question remains open whether this increase in phosphopeptide-binding affinity can be attributed to the tight-bD or loose-bD conformation (or both).

Here, we have briefly investigated this question by docking the phosphopeptide GpYLVLDKW into the sites defined by the pY and pY + 3 pockets of the wild-type and mutant (tight-bD and loose-bD) SH2 domains. SH2db provides easy access to the pre-aligned structures in pdb format, and the pre-assembled Pymol session presents a facile approach for visualizing the structures and binding poses in a unified style and viewpoint, while systematically highlighting the Sheinerman residues (Table 1) that are primarily responsible for phosphotyrosine binding (Figure 4). For docking, we have used the Peptide docking mode of single precision (SP) Glide (49,50), and accepted the best-scored docking pose that presented the characteristic salt bridge between the phosphotyrosine and anchoring arginine R618^bBx50^. The binding pose for the phosphopeptide against the tight-bD conformation is overall quite similar to the one against the wild-type SH2 domain, with part of the peptide reaching over the central β-sheet and into the pY + 3 pocket. By contrast, in the loose-bD conformation, the bD strand forms a small subpocket with the neighbouring loops that can accommodate the N-terminal end of the phospho-peptide. This difference is also reflected in the superior docking score of this pose (-6.029 vs. -3.152 and -2.188 in the tight-bD and wild-type structures respectively, the smaller the better). Based on this brief analysis, we can propose the loose-bD conformation to be primarily responsible for the increased phosphopeptide-binding affinity of the STAT5B^N642H^ SH2 domain. The SH2db webserver greatly facilitated this investigation by providing a convenient starting point to the calculation and visualization within a few clicks.

**Table 1.** Mapping of the newly introduced generic residue numbers against the ‘legacy’ positions of key phosphotyrosine-binding (Sheinerman) residues in STAT5B

Generic residue number	Legacy position	Example (STAT5B)
aAx43	αA2	Lys600
aAx47	αA6	His604
bBx50	βB5	Arg618
bBx52	βB7	Ser620
bBbCx49	bBbC1	Asp621
bBbCx50	bBbC2	Ser622
bDx50	βD4	Asn642
bDx52	βD6	Met644

The dataset also provides predictive power in facilitating functional extrapolations of newly/currently identified mutations (such as those identified from tumour-biopsied patient samples) without structural data. For example, the second most frequent mutation in STAT5B (Y665F) represents a drastic change in polarity, and leads to aggressive leukemias.

By exploring the structural aspects in the context of the SH2 domains, this point mutation (and the loss of the hydroxyl group) reverts the residue to similar hydrophobic residues that are found at the same position in other SH2 domains that have higher peptide affinity. Understanding the structural impacts of mutations can provide information on the phenotype but also whether a specific drug candidate could have potential in the relevant cancer model.

### Case study 2: blocking loop of the N-SH2 domain of the SHP2 phosphatase

**Figure 5.**
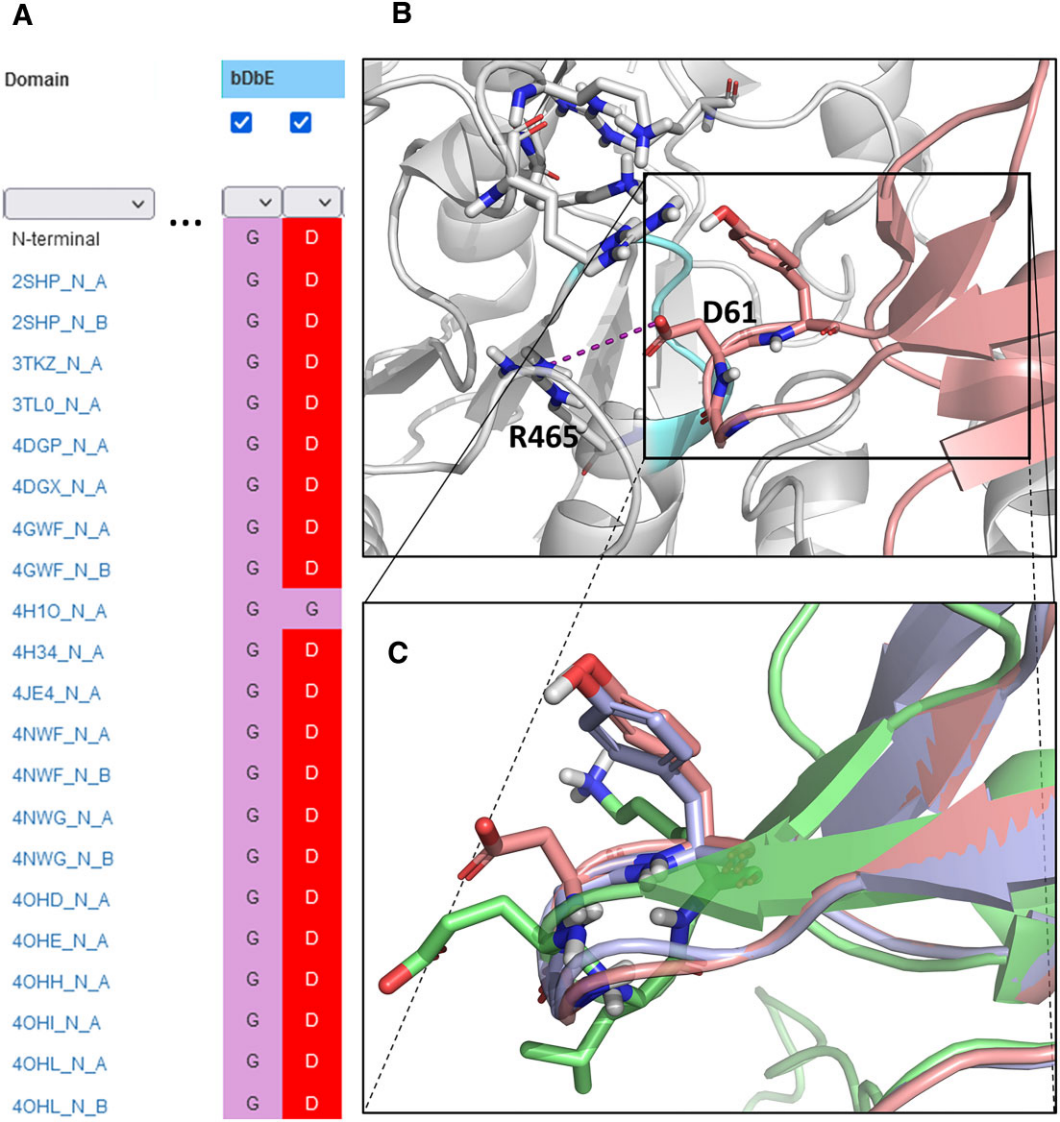
(**A**) Excerpt from the multiple sequence viewer on the Search page: the D61G mutant quickly stands out from the large number of available SHP2 structures. (**B**) Structural requirements for SH2-PTP binding (PDB structure 6BMW (59)): in the closed conformation, the D61 sidechain of the N-SH2 domain (red) bDbE loop can establish a salt bridge with the R465 residue of the PTP domain (white), allowing the N-SH2 domain to block access to the so-called ‘phosphate cradle’ of the active site (cyan). The Y62^bE.48^ sidechain was proposed to interact with the hydrophobic part of the pocket; alternatively, if deprotonated, it could form an additional salt bridge with the proximal cluster of positively charged residues of the PTP domain (highlighted as sticks). (**C**) Compared to the wild-type N-SH2 domain (red), the oncogenic D61G mutant (blue, PDB structure 4H1O, https://www.rcsb.org/structure/4H1O) misses the crucial negatively charged sidechain and is thus not able to form the anchoring salt bridge, resulting in a loss of auto-regulation (permanently active state). The C-SH2 domain (green) has no affinity to the PTP domain either, due in part to the bulkier residues of the bDbE loop (E176, L177), and also to the positively charged sidechain (K178) in the bEx48 position, which should be repulsed by the proximal lysine/arginine cluster.

SH2 domain containing, protein tyrosine phosphatase 2 (SHP2), is a PTP encoded by the PTPN11 gene, which contains two SH2 domains and a protein tyrosine phosphatase (PTP) domain, and has been identified as a pharmaceutical target for a range of oncological indications (51). SHP2 is a prime example of the versatile and diverse utility of SH2 domains, as its phosphatase activity is regulated by an intricate mechanism, where its SH2 domains play central roles, both in conventional and unconventional modes (52,53). Briefly, SHP2 can assume an active and an inactive conformation: in the inactive conformation, the N-terminal SH2 domain closes upon the phosphatase domain, thereby blocking access to its active site. In this atypical regulatory role, the short loop that connects the bD and bE strands of the N-terminal SH2 domain (‘blocking loop’ or bDbE loop following our nomenclature) inserts into the active site of the phosphatase domain, making it inaccessible for substrates (Figure 5B). To release this autoregulatory lock, the N-terminal and C-terminal SH2 domains can simultaneously bind bis-phosphotyrosyl proteins or peptides like IRS-1 (ie. the ‘conventional’ mode of phosphopeptide recognition by SH2 domains), which disrupts the SH2-PTP interaction, allowing access to the active site in an open conformation of the protein (54).

There are several oncogenic mutations that circumvent this autoregulatory lock by stabilizing the active (open) conformation, most notably by weakening/abolishing the SH2-PTP interaction (55). Current pharmaceutical strategies targeting SHP2 are aiming at the stabilization of the closed conformation by small molecular ligands that bind to one of the allosteric sites at the interface of the N-SH2, C-SH2 and PTP domains (labelled ‘tunnel’, ‘latch’ and ‘groove’) (56), or by a combination of such ligands (57).

Here, we demonstrate the utility of SH2db in understanding the structural requirements for the SH2-PTP interaction. From previous studies, it is known that the short blocking loop, or bDbE loop, and the tyrosine residue in the first position of the following bE strand (Y62^bEx48^) are directly involved in binding to the active site of the PTP domain (56). With the interactive sequence viewer on the Search page, we can quickly observe that there is a PDB structure (4H1O) available for the SHP2 mutant D61G (https://www.rcsb.org/structure/4H1O), where the aspartate residue of the blocking loop is replaced by a glycine (Figure 5A): this is an oncogenic mutation that was identified in multiple disease conditions, including Noonan syndrome and leukemia (58,59). With a few clicks, we can download this structure into a Pymol session, with the crucial residues highlighted. As a basis for comparison, we have also included the N-terminal SH2 domain of the wild-type SHP2 from a recent structure (6BMW), as well as the C-terminal SH2 domain from the same structure (57). Our comparison clearly verifies some of the crucial recognition features of the N-SH2 blocking loop (Figure 5C): for example, removal of the acidic D61 sidechain in the oncogenic D61G mutant stabilizes the open (active) conformation of the enzyme by abolishing the ability of the bDbE loop to form a crucial salt bridge with the R465 sidechain upon the closure of the N-SH2 domain onto the PTP domain. In the meantime, the C-SH2 domain should have poor affinity to the PTP domain, due to a number of structural differences, including bulkier residues in its bDbE loop, as well as its different overall fold. In this scenario, it ultimately took very little effort to find the relevant structures and produce a useful visualization to understand the structural requirements of SHP2 autoregulation.

## CONCLUSION AND OUTLOOK

We created an online webserver and database for the SH2 domain containing proteins with a focus on protein sequence and structural data. With the development of the SH2 generic numbering system, we obtained a structure-based alignment for the whole family enabling overall and local comparisons to be easily accessed between different protein family members. The webserver offers a search and browse option through the stored protein and structure data and highlights mutations in the structures. As shown with our two case studies, using the alignment view and downloadable Pymol session, users can quickly identify and navigate to areas of interest within the SH2 domain.

This utility can be expanded to broader queries including mutational and structural predictions for functional analysis. This would empower drug discovery as well as drug candidate forecasting for uncharacterized SH2 mutations that can arise in different patient cancers/diseases. Moreover, protein engineering or upcoming proteomics approaches that leverage SH2 domains or superbinders for pTyr enrichment can benefit from wider SH2 domain analysis. Additionally, the portfolios of alignments and structural data will allow for deeper analysis in comparative genomics between different species and other biotechnological applications.

We aim to update SH2db every six months with newly published structures and new AlphaFold models. In the future, we plan to expand the database to incorporate species orthologs while simultaneously developing new data derived tools for the website.

## DATA AVAILABILITY

All structural and sequence data are made available via the website http://sh2db.ttk.hu. The source code of SH2db is shared via Github at https://github.com/keserulab/SH2db.

## References

[1] Koch C. , Anderson D., Moran M., Ellis C., Pawson T. SH2 and SH3 domains: elements that control interactions of cytoplasmic signaling proteins. Science. 1991; 252:668–674. 1708916 10.1126/science.1708916

[2] Pincus D. , Letunic I., Bork P., Lim W.A. Evolution of the phospho-tyrosine signaling machinery in premetazoan lineages. Proc. Natl. Acad. Sci. U.S.A. 2008; 105:9680–9684. 18599463 10.1073/pnas.0803161105 PMC2443182

[3] Lim W.A. , Pawson T. Phosphotyrosine signaling: evolving a new cellular communication system. Cell. 2010; 142:661–667. 20813250 10.1016/j.cell.2010.08.023 PMC2950826

[4] Pawson T. , Gish G.D., Nash P. SH2 domains, interaction modules and cellular wiring. Trends Cell Biol. 2001; 11:504–511. 11719057 10.1016/s0962-8924(01)02154-7

[5] Diop A. , Santorelli D., Malagrinò F., Nardella C., Pennacchietti V., Pagano L., Marcocci L., Pietrangeli P., Gianni S., Toto A. SH2 Domains: folding, binding and therapeutical approaches. Int. J. Mol. Sci. 2022; 23:15944. 36555586 10.3390/ijms232415944 PMC9783222

[6] Machida K. , Mayer B.J. The SH2 domain: versatile signaling module and pharmaceutical target. Biochim. Biophys. Acta Proteins Proteom. 2005; 1747:1–25. 10.1016/j.bbapap.2004.10.005 15680235

[7] Bradshaw J.M. , Waksman G. Molecular recognition by SH2 domains. Adv. Protein Chem. 2002; 61:161–210. 12461824 10.1016/s0065-3233(02)61005-8

[8] Lappalainen I. , Thusberg J., Shen B., Vihinen M. Genome wide analysis of pathogenic SH2 domain mutations. Proteins: Struct. Funct. Genet. 2008; 72:779–792. 18260110 10.1002/prot.21970

[9] Filippakopoulos P. , Müller S., Knapp S. SH2 domains: modulators of nonreceptor tyrosine kinase activity. Curr. Opin. Struct. Biol. 2009; 19:643–649. 19926274 10.1016/j.sbi.2009.10.001 PMC2791838

[10] de Araujo E.D. , Orlova A., Neubauer H.A., Bajusz D., Seo H.-S., Dhe-Paganon S., Keserű G.M., Moriggl R., Gunning P.T. Structural implications of STAT3 and STAT5 SH2 domain mutations. Cancers (Basel). 2019; 11:1757. 31717342 10.3390/cancers11111757 PMC6895964

[11] Li X. , Lau A.Y.T., Ng A.S.N., Aldehaiman A., Zhou Y., Ng P.K.S., Arold S.T., Cheung L.W.T. Cancer-associated mutations in the p85α N-terminal SH2 domain activate a spectrum of receptor tyrosine kinases. Proc. Natl. Acad. Sci. U.S.A. 2021; 118:e2101751118. 34507989 10.1073/pnas.2101751118 PMC8449365

[12] Machida K. , Liu B. A. SH2 Domains:Methods and Protocols. 2017; New York, NY, USA Humana Press.

[13] Gao Q. , Hua J., Kimura R., Headd J.J., Fu X.Y., Chin Y.E. Identification of the linker-SH2 domain of STAT as the origin of the SH2 domain using two-dimensional structural alignment. Mol. Cell. Proteomics. 2004; 3:704–714. 15073273 10.1074/mcp.M300131-MCP200

[14] Songyang Z. , Cantley L.C. Recognition and specificity in protein tyrosine kinase-mediated signalling. Trends Biochem. Sci. 1995; 20:470–475. 8578591 10.1016/s0968-0004(00)89103-3

[15] Liu B.A. , Jablonowski K., Raina M., Arcé M., Pawson T., Nash P.D. The Human and mouse complement of SH2 domain proteins—establishing the boundaries of phosphotyrosine signaling. Mol. Cell. 2006; 22:851–868. 16793553 10.1016/j.molcel.2006.06.001

[16] Bajusz D. , Miranda-Quintana R.A., Rácz A., Héberger K. Extended many-item similarity indices for sets of nucleotide and protein sequences. Comput Struct Biotechnol J. 2021; 19:3628–3639. 34257841 10.1016/j.csbj.2021.06.021 PMC8253954

[17] Liu B.A. , Engelmann B.W., Nash P.D. The language of SH2 domain interactions defines phosphotyrosine-mediated signal transduction. FEBS Lett. 2012; 586:2597–2605. 22569091 10.1016/j.febslet.2012.04.054

[18] Sheinerman F.B. , Al-Lazikani B., Honig B. Sequence, structure and energetic determinants of phosphopeptide selectivity of SH2 domains. J. Mol. Biol. 2003; 334:823–841. 14636606 10.1016/j.jmb.2003.09.075

[19] Campbell S.J. , Jackson R.M. Diversity in the SH2 domain family phosphotyrosyl peptide binding site. Protein Eng. Des. Sel. 2003; 16:217–227. 10.1093/proeng/gzg025 12702802

[20] Jaber Chehayeb R. , Boggon T.J. SH2 Domain binding: diverse flvrs of partnership. Front. Endocrinol. (Lausanne). 2020; 11:575220. 33042028 10.3389/fendo.2020.575220 PMC7530234

[21] Diallo M. , Herrera F. The role of understudied post-translational modifications for the behavior and function of Signal transducer and activator of transcription 3. FEBS J. 2022; 289:6235–6255. 34235865 10.1111/febs.16116

[22] De Oliveira G.A.P. , Arruda H.R.S., De Andrade G.C., Silva J.L. Evolutionary role of water-accessible cavities in Src homology 2 (SH2) domains. J. Phys. Chem. B. 2022; 126:8689–8698. 36281877 10.1021/acs.jpcb.2c05409

[23] Martyn G.D. , Veggiani G., Kusebauch U., Morrone S.R., Yates B.P., Singer A.U., Tong J., Manczyk N., Gish G., Sun Z. et al. Engineered SH2 domains for targeted phosphoproteomics. ACS Chem. Biol. 2022; 17:1472–1484. 35613471 10.1021/acschembio.2c00051 PMC9251651

[24] Stiegler A.L. , Vish K.J., Boggon T.J. Tandem engagement of phosphotyrosines by the dual SH2 domains of p120RasGAP. Structure. 2022; 30:1603–1614. 36417908 10.1016/j.str.2022.10.009 PMC9722645

[25] Isberg V. , Vroling B., Van Der Kant R., Li K., Vriend G., Gloriam D. GPCRDB: an information system for G protein-coupled receptors. Nucleic Acids Res. 2014; 42:D422–D425. 24304901 10.1093/nar/gkt1255 PMC3965068

[26] Isberg V. , de Graaf C., Bortolato A., Cherezov V., Katritch V., Marshall F.H., Mordalski S., Pin J.-P., Stevens R.C., Vriend G. et al. Generic GPCR residue numbers – aligning topology maps while minding the gaps. Trends Pharmacol. Sci. 2015; 36:22–31. 25541108 10.1016/j.tips.2014.11.001 PMC4408928

[27] Munk C. , Harpsøe K., Hauser A.S., Isberg V., Gloriam D.E. Integrating structural and mutagenesis data to elucidate GPCR ligand binding. Curr. Opin. Pharmacol. 2016; 30:51–58. 27475047 10.1016/j.coph.2016.07.003 PMC6910865

[28] Pándy-Szekeres G. , Munk C., Tsonkov T.M., Mordalski S., Harpsøe K., Hauser A.S., Bojarski A.J., Gloriam D.E. GPCRdb in 2018: adding GPCR structure models and ligands. Nucleic Acids Res. 2018; 46:D440–D446. 29155946 10.1093/nar/gkx1109 PMC5753179

[29] Jumper J. , Evans R., Pritzel A., Green T., Figurnov M., Ronneberger O., Tunyasuvunakool K., Bates R., Žídek A., Potapenko A. et al. Highly accurate protein structure prediction with AlphaFold. Nature. 2021; 596:583–589. 34265844 10.1038/s41586-021-03819-2 PMC8371605

[30] Pándy-Szekeres G. , Caroli J., Mamyrbekov A., Kermani A.A., Keserű G.M., Kooistra A.J., Gloriam D.E. GPCRdb in 2023: state-specific structure models using AlphaFold2 and new ligand resources. Nucleic Acids Res. 2023; 51:D395–D402. 36395823 10.1093/nar/gkac1013 PMC9825476

[31] van Linden O.P.J. , Kooistra A.J., Leurs R., de Esch I.J.P., de Graaf C. KLIFS: a knowledge-based structural database to navigate kinase-ligand interaction space. J. Med. Chem. 2014; 57:249–277. 23941661 10.1021/jm400378w

[32] Kanev G.K. , de Graaf C., Westerman B.A., de Esch I.J.P., Kooistra A.J. KLIFS: an overhaul after the first 5 years of supporting kinase research. Nucleic Acids Res. 2021; 49:D562–D569. 33084889 10.1093/nar/gkaa895 PMC7778968

[33] Diella F. , Cameron S., Gemünd C., Linding R., Via A., Kuster B., Sicheritz-Pontén T., Blom N., Gibson T.J. Phospho.ELM: a database of experimentally verified phosphorylation sites in eukaryotic proteins. BMC Bioinf. 2004; 5:79. 10.1186/1471-2105-5-79 PMC449700 15212693

[34] Dinkel H. , Chica C., Via A., Gould C.M., Jensen L.J., Gibson T.J., Diella F. Phospho.ELM: a database of phosphorylation sites-update 2011. Nucleic Acids Res. 2011; 39:D261–D267. 21062810 10.1093/nar/gkq1104 PMC3013696

[35] Obenauer J.C. , Cantley L.C., Yaffe M.B. Scansite 2.0: proteome-wide prediction of cell signalling interactions using short sequence motifs. Nucleic Acids Res. 2003; 31:3635–3641. 12824383 10.1093/nar/gkg584 PMC168990

[36] Berman H.M. , Westbrook J., Feng Z., Gilliland G., Bhat T.N., Weissig H., Shindyalov I.N., Bourne P.E. The Protein Data Bank. Nucleic Acids Res. 2000; 28:235–242. 10592235 10.1093/nar/28.1.235 PMC102472

[37] Tunyasuvunakool K. , Adler J., Wu Z., Green T., Zielinski M., Žídek A., Bridgland A., Cowie A., Meyer C., Laydon A. et al. Highly accurate protein structure prediction for the human proteome. Nature. 2021; 596:590–596. 34293799 10.1038/s41586-021-03828-1 PMC8387240

[38] The Uniprot Consortium UniProt: the universal protein knowledgebase in 2021. Nucleic Acids Res. 2021; 49:D480–D489. 33237286 10.1093/nar/gkaa1100 PMC7778908

[39] Berman H. , Henrick K., Nakamura H. Announcing the worldwide Protein Data Bank. Nat. Struct. Biol. 2003; 10:980. 14634627 10.1038/nsb1203-980

[40] Varadi M. , Anyango S., Deshpande M., Nair S., Natassia C., Yordanova G., Yuan D., Stroe O., Wood G., Laydon A. et al. AlphaFold Protein Structure Database: massively expanding the structural coverage of protein-sequence space with high-accuracy models. Nucleic Acids Res. 2022; 50:D439–D444. 34791371 10.1093/nar/gkab1061 PMC8728224

[41] Ballesteros J.A. , Weinstein H. Integrated methods for the construction of three-dimensional models and computational probing of structure-function relations in G protein-coupled receptors. Methods Neurosci. 1995; 25:366–428.

[42] Wingelhofer B. , Neubauer H.A., Valent P., Han X., Constantinescu S.N., Gunning P.T., Müller M., Moriggl R. Implications of STAT3 and STAT5 signaling on gene regulation and chromatin remodeling in hematopoietic cancer. Leukemia. 2018; 32:1713–1726. 29728695 10.1038/s41375-018-0117-x PMC6087715

[43] Yu H. , Jove R. The stats of cancer - new molecular targets come of age. Nat. Rev. Cancer. 2004; 4:97–105. 14964307 10.1038/nrc1275

[44] Orlova A. , Wingelhofer B., Neubauer H.A., Maurer B., Berger-Becvar A., Keserű G.M., Gunning P.T., Valent P., Moriggl R. Emerging therapeutic targets in myeloproliferative neoplasms and peripheral T-cell leukemia and lymphomas. Expert Opin. Ther. Targets. 2018; 22:45–57. 29148847 10.1080/14728222.2018.1406924 PMC5743003

[45] Wingelhofer B. , Maurer B., Heyes E.C., Cumaraswamy A.A., Berger-Becvar A., de Araujo E.D., Orlova A., Freund P., Ruge F., Park J. et al. Pharmacologic inhibition of STAT5 in acute myeloid leukemia. Leukemia. 2018; 32:1135–1146. 29472718 10.1038/s41375-017-0005-9 PMC5940656

[46] Orlova A. , Wagner C., de Araujo E.D., Bajusz D., Neubauer H.A., Herling M., Gunning P.T., Keserű G.M., Moriggl R. Direct targeting options for STAT3 and STAT5 in cancer. Cancers (Basel). 2019; 11:1930. 31817042 10.3390/cancers11121930 PMC6966570

[47] de Araujo E.D. , Erdogan F., Neubauer H.A., Meneksedag-Erol D., Manaswiyoungkul P., Eram M.S., Seo H.-S., Qadree A.K., Israelian J., Orlova A. et al. Structural and functional consequences of the STAT5BN642H driver mutation. Nat. Commun. 2019; 10:2517. 31175292 10.1038/s41467-019-10422-7 PMC6555848

[48] Müller J. , Schust J., Berg T. A high-throughput assay for signal transducer and activator of transcription 5b based on fluorescence polarization. Anal. Biochem. 2008; 375:249–254. 18258175 10.1016/j.ab.2008.01.017

[49] Friesner R.A. , Banks J.L., Murphy R.B., Halgren T.A., Klicic J.J., Mainz D.T., Repasky M.P., Knoll E.H., Shelley M., Perry J.K. et al. Glide: a new approach for rapid, accurate docking and scoring. 1. Method and assessment of docking accuracy. J. Med. Chem. 2004; 47:1739–1749. 15027865 10.1021/jm0306430

[50] Halgren T.A. , Murphy R.B., Friesner R.A., Beard H.S., Frye L.L., Pollard W.T., Banks J.L. Glide: a new approach for rapid, accurate docking and scoring. 2. Enrichment factors in database screening. J. Med. Chem. 2004; 47:1750–1759. 15027866 10.1021/jm030644s

[51] Song Y. , Zhao M., Zhang H., Yu B. Double-edged roles of protein tyrosine phosphatase SHP2 in cancer and its inhibitors in clinical trials. Pharmacol. Ther. 2022; 230:107966. 34403682 10.1016/j.pharmthera.2021.107966

[52] Hof P. , Pluskey S., Dhe-Paganon S., Eck M.J., Shoelson S.E. Crystal structure of the tyrosine phosphatase SHP-2. Cell. 1998; 92:441–450. 9491886 10.1016/s0092-8674(00)80938-1

[53] Barford D. , Neel B.G. Revealing mechanisms for SH2 domain mediated regulation of the protein tyrosine phosphatase SHP-2. Structure. 1998; 6:249–254. 9551546 10.1016/s0969-2126(98)00027-6

[54] Pluskey S. , Wandless T.J., Walsh C.T., Shoelson S.E. Potent stimulation of SH-PTP2 phosphatase activity by simultaneous occupancy of both SH2 domains. J. Biol. Chem. 1995; 270:2897–2900. 7531695 10.1074/jbc.270.7.2897

[55] LaRochelle J.R. , Fodor M., Vemulapalli V., Mohseni M., Wang P., Stams T., LaMarche M.J., Chopra R., Acker M.G., Blacklow S.C. Structural reorganization of SHP2 by oncogenic mutations and implications for oncoprotein resistance to allosteric inhibition. Nat. Commun. 2018; 9:4508. 30375388 10.1038/s41467-018-06823-9 PMC6207684

[56] Lamarche M.J. , Acker M., Argintaru A., Bauer D., Boisclair J., Chan H., Chen C.H.T., Chen Y.N., Chen Z., Deng Z. et al. Identification of TNO155, an allosteric SHP2 inhibitor for the treatment of cancer. J. Med. Chem. 2020; 63:13578–13594. 32910655 10.1021/acs.jmedchem.0c01170

[57] Fodor M. , Price E., Wang P., Lu H., Argintaru A., Chen Z., Glick M., Hao H.X., Kato M., Koenig R. et al. Dual allosteric inhibition of SHP2 phosphatase. ACS Chem. Biol. 2018; 13:647–656. 29304282 10.1021/acschembio.7b00980

[58] Kratz C.P. , Niemeyer C.M., Castleberry R.P., Cetin M., Bergsträsser E., Emanuel P.D., Hasle H., Kardos G., Klein C., Kojima S. et al. The mutational spectrum of PTPN11 in juvenile myelomonocytic leukemia and Noonan syndrome/myeloproliferative disease. Blood. 2005; 106:2183–2185. 15928039 10.1182/blood-2005-02-0531 PMC1895140

[59] Bobone S. , Pannone L., Biondi B., Solman M., Flex E., Canale V.C., Calligari P., De Faveri C., Gandini T., Quercioli A. et al. Targeting oncogenic src homology 2 domain-containing phosphatase 2 (SHP2) by inhibiting its protein-protein interactions. J. Med. Chem. 2021; 64:15973–15990. 34714648 10.1021/acs.jmedchem.1c01371 PMC8591604

[60] Letunic I. , Bork P. Interactive Tree Of Life (iTOL) v5: an online tool for phylogenetic tree display and annotation. Nucleic Acids Res. 2021; 49:W293–W296. 33885785 10.1093/nar/gkab301 PMC8265157

